# Dr. Ferdinand J. S. Gorgas, A.B., A.M., D.D.S., M.D.

**Published:** 1914-11-15

**Authors:** 


### OBITUARY.

Dr. Ferdinand J. S. Gorgas, A.B., A.M., D.D.S., M.D.—an honorable fellow of the American Academy of Dental Science, died in Baltimore, Md., April 8th, 1914, in the eightieth (80th) year of his life. In his profession he was known and honored as one of the most notable men of his time, both as author and educator. He received the degrees of A.B. and A.M., from Dickerson College, and his dental degree in 1854 from Baltimore Dental College where he taught, as Dean and Professor from 1867 until 1882. He received his medical degree from the University of Maryland in 1863 and was Dean and Professor there from 1882 until 1911, where he held the chairs of Prosthetic Dentistry, Oral Surgery, and Dental Medicine. He was editor of the "American Journal of Dental Surgery", of "Harris' Principals and Practice of Dentistry" and of "Harris' Dental Dictionary". He was also author of a work on "Dental Medicine" and of "Questions and Answers for Dental Students".

He was a member of the Maryland State Dental Association, and an honorary fellow of many societies. His burial was at Greenmout cemetery, Baltimore. Professor Gorgas was an enthusiastic teacher, ever eager to raise the ethical and education standard of his profession. A wife and two sons survive him. These sons are Dr. L. D. Gorgas of Chicago, Ills., and Dr. H. F. Gorgas of Baltimore, Md.

RESOLVED: That in the death of Professor Gorgas, the Academy has lost one of its most valued fellows, whose colleagues, recognizing his great educational power, gave to him the many important positions which he held with such marked ability in the Institutions he so faithfully served.

Respectfully submitted,

ROBERT R. ANDREWS,
FORREST G. EDDY,
EDWARD C. BRIGGS,
Committee.

